# Monocytes and the Host Response to Fungal Pathogens

**DOI:** 10.3389/fcimb.2020.00034

**Published:** 2020-02-13

**Authors:** Lena J. Heung

**Affiliations:** Division of Infectious Diseases, Department of Medicine, Cedars-Sinai Medical Center, Los Angeles, CA, United States

**Keywords:** monocyte, macrophage, dendritic cell, fungal infections, innate immunity

## Abstract

Monocytes and their derivatives, including macrophages and dendritic cells, play diverse roles in the response to fungal pathogens. Sensing of fungi by monocytes triggers signaling pathways that mediate direct effects like phagocytosis and cytokine production. Monocytes can also present fungal antigens to elicit adaptive immune responses. These monocyte-mediated pathways may be either beneficial or harmful to the host. In some instances, fungi have developed mechanisms to evade the consequences of monocyte activation and subvert these cells to promote disease. Thus, monocytes are critically involved in mediating the outcomes of these often highly fatal infections. This review will highlight the roles of monocytes in the immune response to some of the major fungi that cause invasive human disease, including *Aspergillus, Cryptococcus, Candida, Histoplasma, Blastomyces*, and *Coccidioides*, and discuss potential strategies to manipulate monocyte responses in order to enhance anti-fungal immunity in susceptible hosts.

## Introduction

**Figure 1:**
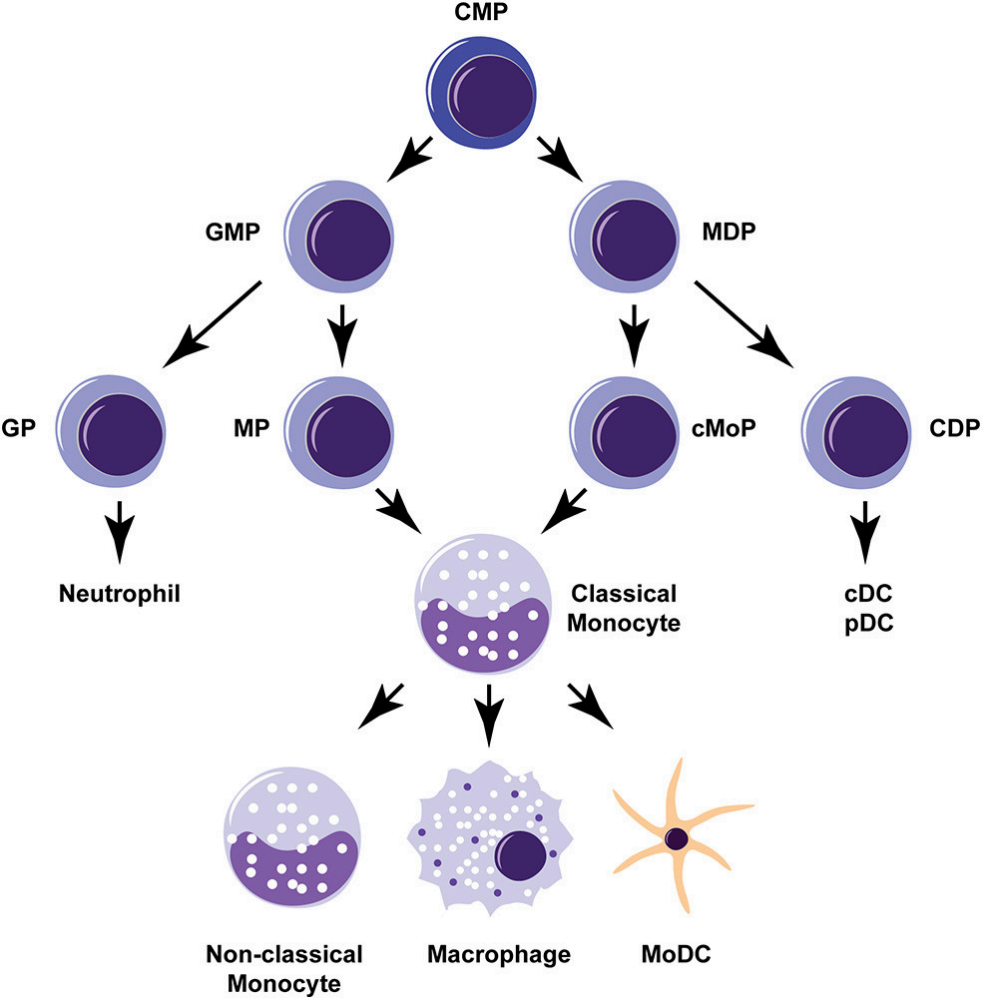
Monocyte development and differentiation pathways. Monocytes can develop from either a granulocyte-monocyte progenitor (GMP) or a monocyte-dendritic cell progenitor (MDP). Classical “inflammatory” monocytes can give rise to non-classical “patrolling” monocytes or further differentiate into macrophages or monocyte-derived dendritic cells (MoDCs). Common myeloid progenitor (CMP), granulocyte progenitor (GP), monocyte-committed progenitor (MP), common monocyte progenitor (cMoP), common dendritic cell progenitor (CDP), conventional dendritic cell (cDC), plasmacytoid dendritic cell (pDC).

Monocytes are innate immune cells that may be generated in the bone marrow from two different precursors, either a granulocyte-monocyte progenitor (GMP) or a monocyte-dendritic cell progenitor (MDP) (Yanez et al., 2017; Wolf et al., 2019; Figure 1). Monocytes derived from either of these lineages consist of two main types: (1) classical “inflammatory” monocytes that are CCR2^+^ Ly6C^hi^ in mice and CD14^+^ CD16^−^ in humans, and (2) non-classical “patrolling” monocytes that are CCR2^lo^ Ly6C^lo^ in mice and CD14^lo^ CD16^+^ in humans. Lineage tracing studies suggest that non-classical monocytes develop directly from classical monocytes (Yona et al., 2013). During homeostatic conditions, non-classical monocytes patrol the circulation to engage in tissue repair and clearance of dead cells (Auffray et al., 2007). During inflammation or infection, classical monocytes are mobilized from bone marrow reserves in response to chemokines that bind to the CCR2 receptor, such as CCL2 and CCL7 (Shi and Pamer, 2011). Upon entering affected tissues, classical monocytes can further differentiate into macrophages and monocyte-derived dendritic cells (MoDCs). Macrophages that differentiate from monocytes in the adult bone marrow are distinguished from tissue resident macrophages (e.g., alveolar macrophages, glial cells) that originally derive from fetal yolk sac progenitor cells or monocytes from the fetal liver (Hoeffel and Ginhoux, 2018). However, there is evidence that bone marrow-derived monocytes can help replenish the tissue-resident macrophages of specific organs including the gut, the skin, and the heart (Ginhoux and Guilliams, 2016). MoDCs are also distinct in origin from conventional dendritic cells (cDCs) and plasmacytoid dendritic cells (pDCs) that are derived from a common dendritic cell progenitor (CDP). The ontogeny of monocytes and ongoing controversies about their origins and development are reviewed in further detail elsewhere (Jakubzick et al., 2017; Murray, 2018; Wolf et al., 2019).

Invasive fungal infections represent a significant cause of human disease, with an estimated 1.5 million people dying each year (Bongomin et al., 2017). Unfortunately, the incidence of these infections is increasing with the expanded use of immunosuppressive therapies, broad-spectrum antibiotics, and invasive medical devices (Pfaller and Diekema, 2010; Clark and Drummond, 2019). Invasive infections are commonly caused by fungi of the genera *Aspergillus, Candida, Cryptococcus, Blastomyces, Coccidioides*, and *Histoplasma* (Table 1). Except for *Candida* species, which are commensal organisms found on the skin and mucosal surfaces, these fungi are environmental microorganisms that are typically acquired after inhalation into the lungs. All of these fungi can cause invasive disease in a wide-spectrum of immunocompromised patients, such as those with genetic immunodeficiencies, HIV/AIDS, cancer, solid organ, and hematopoietic stem cell transplantation, autoimmune diseases, immunosuppressive treatments, and other predisposing states like diabetes and pregnancy. However, apparently immunocompetent patients can also be affected by many of these fungi, including *Candida, Cryptococcus gattii*, and the endemic fungi *Blastomyces, Coccidioides*, and *Histoplasma*. Unfortunately, the morbidity and mortality from invasive fungal infections remains quite high despite current drug regimens, some of which utilize multiple antifungal agents. Therefore, the development of novel therapeutic approaches to fungal infections is imperative to improving clinical outcomes.

**Table 1:** Fungal organisms that induce monocyte-mediated responses.

	**Organism**	**Typical clinical presentations**	**Key roles of monocytes and derivatives**
Mold	*Aspergillus* spp. (*A. fumigatus, A. terreus*)	Pneumonia and other respiratory tract infections, systemic infections in immunocompromised patients	**Beneficial:** Phagocytize and kill conidia, cytokine production to enhance fungicidal activity of neutrophils, transport conidia to the lymph nodes and facilitate adaptive CD4^+^ T cell responses **Harmful:** Reservoir of viable conidia
Yeast*	*Cryptococcus* spp. (*C. neoformans, C. gattii*)	Pneumonia, meningitis in immunocompromised patients and apparently immunocompetent patients (*C. gattii*)	**Beneficial:** Phagocytize and kill conidia, T cell priming **Harmful:** Reservoir of viable yeast that aids in dissemination
Polymorphic fungus	*Candida albicans*	Blood stream infections, deep infections often related to medical devices or surgical interventions, disseminated disease, skin and mucosal infections	**Beneficial:** Cytokine production and cellular crosstalk with NK cells to enhance fungicidal activity of neutrophils, elicit Th1 and Th17 immune responses, innate immune memory
Dimorphic fungi	*Blastomyces dermatitidis*	Pneumonia, disseminated disease	**Beneficial:** T cell priming **Harmful:** Reservoir in which spores convert into proliferative yeast forms
	*Coccidioides* spp. (*C. immitis, C. posadasii)*	Pneumonia, meningitis, disseminated disease	**Beneficial:** Phagocytize and kill arthroconidia and endospores, pro-inflammatory cytokine production
	*Histoplasma capsulatum*	Pneumonia, disseminated disease	**Beneficial:** Phagocytize and kill yeast, antigen processing/presentation to T cells **Harmful:** Reservoir of viable yeast (resting macrophages and monocytes)

* *Exhibits filamentous growth in the environment during the mating cycle*.

Monocytes and their derivatives have been found to play critical roles in the outcomes of fungal infections (Table 1). For example, monocyte-deficient mice are more susceptible to infections with *Aspergillus fumigatus* (Hohl et al., 2009; Espinosa et al., 2014), *Candida albicans* (Ngo et al., 2014; Dominguez-Andres et al., 2017b), and *Histoplasma capsulatum* (Szymczak and Deepe, 2009). On the other hand, the absence of monocytes during *Cryptococcus neoformans* infection can either be detrimental or beneficial to host outcomes, depending on the infection model (Traynor et al., 2000; Osterholzer et al., 2008, 2009; Charlier et al., 2009; Heung and Hohl, 2019). This plasticity of monocytes in the regulation of immune responses to fungi makes these cells ideal targets for immunomodulatory therapies. Indeed, strategies to target monocyte development and function are already under investigation as potential cancer therapies given their roles in facilitating both pro-tumor and anti-tumor effects (Olingy et al., 2019). This review will highlight the key mechanisms by which monocytes regulate innate immunity to fungi, including fungal sensing, phagocytosis, cytokine production and cellular crosstalk, and antigen presentation and T cell priming. Recent developments in understanding the role of trained immunity in monocyte responses to fungal pathogens will also be discussed.

## Fungal Sensing and Orchestration of the Immune Response

Monocytes express a variety of receptors to facilitate detection of fungal cells. Pattern recognition receptors (PRRs), including C-type lectin receptors (CLRs), Toll-like receptors (TLRs), and NOD-like receptors (NLRs), can detect pathogen-associated molecular patterns (PAMPs) like β-glucan, chitin and mannose in the fungal cell wall and trigger downstream signaling pathways to coordinate the innate immune response (Lionakis et al., 2017). Complement receptors (CRs) and Fc receptors also assist in fungal sensing by detecting complement or antibody-bound fungal cells (Erwig and Gow, 2016). These receptors can have individual effects or work in collaboration with each other. For example, cytokine production by macrophages and DCs is regulated by the CLR Dectin-1 and complement receptor 3 (CR3) during *H. capsulatum* infection and by Dectin-1 and TLR2 in *Coccidioides* infection models (Viriyakosol et al., 2005, 2013; del Pilar Jimenez et al., 2008; Lin et al., 2010; Huang et al., 2015). The activation of the NLRP3 inflammasome during histoplasmosis is coordinated by Dectin-1 and Dectin-2 signaling (Chang et al., 2017). During *C. albicans* infection, Dectin-1, Dectin-2, and Mincle collectively contribute to host defenses by regulating monocyte cytokine production and phagocytosis of the fungus (Thompson et al., 2019). Additionally, the balance between Dectin-1 and TLR signaling in MoDCs can determine the Th1 and Th17 responses to *A. fumigatus* (Rivera et al., 2011).

An interesting facet of PRR expression by monocytes is the capacity to discern different morphologic forms of fungi. *C. albicans* exists in yeast and filamentous forms, which can be present at different stages of the infection process (Noble et al., 2017). The morphogenesis of *C. albicans* from yeast to hyphae at mucosal surfaces activates the NLRP3 inflammasome in macrophages, which can stimulate Th17 responses that are important for mucosal defense (Joly et al., 2009; Gow et al., 2011). Dectin-1 on macrophages can bind to β-glucan that is exposed at budding sites on the yeast form of *C. albicans* which triggers phagocytosis and reactive oxygen species (ROS) production (Gantner et al., 2005). Additionally, DCs exposed to *C. albicans* yeast can induce Th1 immune responses, while exposure to hyphal forms elicits Th2 responses (d'Ostiani et al., 2000). The mold *A. fumigatus* forms airborne spores called conidia. Under permissive conditions, these resting conidia can be induced to swell, germinate, and form hyphae that can invade underlying tissues. Germination involves shedding of the immunosuppressive outer rodlet layer of conidia and exposure of PAMPs in the fungal cell wall, including β-glucan and α-mannan (Aimanianda et al., 2009). These PAMPs are detected by Dectin-1 and Dectin-2, resulting in the activation of NF-κβ and pro-inflammatory cytokine production by macrophages and moDCs (Hohl et al., 2005; Steele et al., 2005; Gersuk et al., 2006; Carrion Sde et al., 2013). Different receptors also mediate phagocytosis of the different forms of *A. fumigatus*. Mannose receptor can regulate the uptake of conidia by DCs, while FcγRII and FcγRIII assist with uptake of hyphal forms (Bozza et al., 2002). Similar to *C. albicans*, the sensing of different morphotypes of *A. fumigatus* can affect the adaptive immune response. Metabolically active, live *A. fumigatus* conidia induce beneficial Th1 CD4^+^ T cell responses, while inactive, heat-killed conidia and hyphae can stimulate a Th2-skewed response (Bozza et al., 2002; Hohl et al., 2005; Rivera et al., 2005). Thus, the ability to sense different morphologies of fungi may enable monocytes and their derivatives to distinguish potentially invasive forms from non-invasive forms, as well as different stages of fungal infection, so that the immune system can respond accordingly.

## Phagocytosis: Host Or Pathogen Advantage?

Phagocytosis of fungal cells by monocytes and their derivative macrophages and DCs is another key element of the immune response. Fungi can be eliminated in these cells in the phagolysosome, an acidified compartment that can sequester nutrients and contains various enzymes, ROS generated by NADPH oxidase (NOX2), and reactive nitrogen species (RNS) produced by inducible nitric oxide synthase (iNOS or NOS2) in response to pro-inflammatory stimuli (Uribe-Querol and Rosales, 2017). This fungal killing may be sufficient to halt the progression of infection, but it can also provide fungal antigens that can be used to initiate the adaptive immune response to ensure sterilizing immunity. Fungal uptake is not always beneficial to the host, however, as some fungi have adapted to the harsh environment in the phagolysosome or can subvert monocytes to enable fungal persistence and proliferation.

Macrophages are the prototypical phagocyte and are conventionally described as polarizing into either pro-inflammatory, classically-activated (M1) macrophages or anti-inflammatory, alternatively-activated (M2) macrophages (Lawrence and Natoli, 2011). This M1/M2 classification is based on the expression of particular markers. For example, M1 macrophages typically express NOS2. M2 macrophages express markers like transglutaminase 2 (TGM2), arginase 1 (ARG1), resistin-like molecule-alpha (RETNLA/FIZZ1), chitinase-like 3 (CHIL3/YM1), and chitinase-like 4 (CHIL4/Ym2), the latter four being murine-specific. M1 macrophage polarization can be induced by IFNγ, GM-CSF, or lipopolysaccharide (LPS), while M2 polarization can be induced by IL-4 or IL-13. Despite this binary designation, macrophages are actually quite heterogenous along the spectrum from M1 to M2, so other classification schemes have been proposed but have not yet been used consistently in the literature (Mosser and Edwards, 2008; Martinez and Gordon, 2014; Murray et al., 2014).

M1 macrophages are typically fungicidal cells. For example, while *H. capsulatum* can replicate within resting (M0) macrophages and monocytes, activation of these cells with cytokines, including IFNγ and GM-CSF, restricts the intracellular growth of *H. capsulatum*, in part by sequestering nutrients like zinc ions that are needed for fungal growth (Howard, 1964; Wu-Hsieh and Howard, 1987; Newman et al., 1991; Subramanian Vignesh et al., 2013). Similarly, *Coccidioides immitis* arthroconidia (the spore form) can survive within unstimulated macrophages *in vitro*, but the addition of IFNγ or TNF enables fungal killing (Beaman et al., 1983). Human monocytes do have an innate ability to take up and kill *C. immitis* arthroconidia, however killing of endospores (the replicating form within the host) requires stimulation by pro-inflammatory cytokines (Ampel and Galgiani, 1991; Beaman, 1991; Ampel et al., 1992). *A. fumigatus* induces M1 polarization and ROS production by macrophages, and mice that lack NOX2 activity in monocytes and macrophages are highly susceptible to *A. fumigatus* infection (Gersuk et al., 2006; Grimm et al., 2013; Zhang et al., 2019). M1 macrophages are more fungicidal against *C. neoformans* than M2 macrophages *in vitro* (Davis et al., 2013). *In vivo*, a shift in macrophage polarization from M2 to M1 correlates with the fungal clearance phase in a murine model of chronic cryptococcosis, and M1 polarization has been associated with host protection against *C. neoformans* in vaccination models (Osterholzer et al., 2011; Hardison et al., 2012). Interestingly, *C. neoformans* has been able to take advantage of the dynamic process of macrophage polarization. In a fatal infection model of cryptococcosis, the fungus induces monocytes to assume an M2 macrophage phenotype that is permissive for fungal proliferation and dissemination (Heung and Hohl, 2019). However, disrupting IL-4 and IL-13 signaling can improve murine outcomes after *C. neoformans* challenge (Stenzel et al., 2009; Muller et al., 2013).

MoDCs can also have direct fungicidal effects. For instance, MoDCs take up and kill *A. fumigatus* conidia, a process mediated in part by NOX2 (Espinosa et al., 2014). They have been shown to engulf and kill both *C. neoformans* and *C. gattii*, although this leads to different outcomes in the adaptive immune responses to the two species (Wozniak and Levitz, 2008; Huston et al., 2013). Additionally, MoDCs kill and process *H. capsulatum* for subsequent antigen presentation to T cells (Gildea et al., 2001).

Fungi have developed counteractive mechanisms to avoid or survive within the phagolysosome. *Cryptococcus* species produce a large polysaccharide capsule to avoid phagocytosis in the first place, but they also can survive within the phagolysosome of monocytes and macrophages (Feldmesser et al., 2000; Alvarez and Casadevall, 2006; Ma et al., 2006; Alvarez et al., 2008, 2009; Heitman et al., 2010; Nicola et al., 2011). *C. immitis* endospores and arthroconidia have been shown to block the fusion of phagosomes with the lysosome in monocytes and macrophages (Beaman and Holmberg, 1980; Beaman et al., 1981). *H. capsulatum* blocks phagosome-lysosome fusion in macrophages and can inhibit acidification of the phagolysosome (Eissenberg et al., 1993; Newman et al., 1997). *Aspergillus terreus*, which can be more refractory to treatment than other *Aspergillus* species, persists as viable conidia in the phagolysosome of macrophages and MoDCs, in addition to dampening the expression of pro-inflammatory cytokines and markers of transmigration by DCs (Slesiona et al., 2012; Hachem et al., 2014; Hsieh et al., 2017). *B. dermatitidis* spores are readily taken up by lung macrophages, but this step promotes the conversion of spores into the yeast form of the fungus with subsequent proliferation (Sterkel et al., 2015). The yeast form of *B. dermatitidis* has also been found to reduce nitric oxide production by macrophages by inhibiting NOS2 activity (Rocco et al., 2011). These studies clearly indicate that the regulation of phagocytosis and maturation of the phagolysosome in monocytes and their derivatives play key roles in the outcomes of fungal infections and, therefore, may be important targets for enhancing host antifungal immunity.

## Cytokine Production and Cellular Crosstalk

The inflammatory milieu generated by monocyte-derived cytokine and chemokine secretion is important for the development of both the innate and adaptive immune response to fungal pathogens. Human susceptibility to blood stream infections with *Candida* has been correlated to single-nucleotide polymorphisms in monocyte-derived cytokines (Jaeger et al., 2019). Monocytes and their derivative cells can produce pro-inflammatory cytokines like TNF, IL-1, and IL-12, anti-inflammatory cytokines like IL-10 and TGF-β, pleiotropic cytokines like IL-6 and IL-15, and chemokines like CXCL1, CXCL2, CCL5, CXCL9, and CXCL10 (Carson et al., 1995; Arango Duque and Descoteaux, 2014). These molecules can influence the activation and recruitment of other immune cells and the polarization of the adaptive immune response.

The secretion of pro-inflammatory cytokines is typically associated with beneficial host responses. Monocytes and their derivatives are an important source of TNF and other pro-inflammatory cytokines and chemokines during infection with *A. fumigatus* and *C. albicans* (Kim et al., 2005; Cortez et al., 2006; Gersuk et al., 2006; Espinosa et al., 2014). In response to *H. capsulatum*, macrophages and DCs secrete pro-inflammatory TNF, IL-6, and IL-12 that inform the adaptive immune response (Lin et al., 2010; Huang et al., 2015). Anti-inflammatory cytokines are more often correlated with poor antifungal immunity. For instance, during *Coccidioides* infection, the susceptibility of C57BL/6 mice is strongly correlated with IL-10 secretion (Jimenez Mdel et al., 2006; Fierer, 2007). DBA/2 mice that are more resistant to *Coccidioides* have DCs that produce less IL-10 and both macrophages and DCs that produce more pro-inflammatory cytokines compared to C57BL/6 mice (del Pilar Jimenez et al., 2008). Additionally, IL-1RA is secreted by human whole blood samples and murine bone marrow-derived DCs upon stimulation with coccidioidal antigens, and it may play a role in blocking IL-1R1 signaling that is host protective (Ampel et al., 2018; Viriyakosol et al., 2018).

Monocyte-derived cytokines can also mediate crosstalk with other innate effector cells. During systemic *C. albicans* infection, IL-15 secreted by monocytes and IL-23p19 produced by DCs induce natural killer (NK) cells to secrete GM-CSF that is required for neutrophil fungicidal activity (Whitney et al., 2014; Dominguez-Andres et al., 2017b). Additionally, monocytes and moDCs promote the fungicidal activity of neutrophils against *A. fumigatus* conidia through the production of pro-inflammatory cytokines (Espinosa et al., 2014). Therefore, manipulation of cytokines produced by monocytes and their derivative cells can have a significant impact on both the innate and adaptive immune response.

## Antigen Presentation and the Adaptive Immune Response

Monocytes and their derivatives are capable of serving as antigen presenting cells (APCs) to prime T cells and induce adaptive immune responses that promote fungal clearance (Roy and Klein, 2012). DCs are the main professional APCs and can pick up fungal antigens by scavenging apoptotic infected cells and antigens shed by fungal cells or by ingesting and processing fungal pathogens directly (Bozza et al., 2002; Lin et al., 2005). After antigen acquisition, DCs mature, as evidenced by upregulation of surface markers including major histocompatibility complex I (MHC I) and II (MHCII) molecules that present antigen to CD8^+^ and CD4^+^ T cells, respectively, and co-stimulatory molecules such as CD80 and CD86. The mature DCs then migrate to lymphoid tissues where they encounter and prime T cells (Eisenbarth, 2019).

In a murine model of chronic cryptococcosis, monocytes can differentiate into DCs that mediate the generation of a Th1 adaptive response that aids in clearance of the fungus (Osterholzer et al., 2009). DCs have been shown to ingest *C. neoformans* or bind cryptococcal antigens, resulting in DC maturation and the subsequent activation and proliferation of T cells (Syme et al., 2002; Mansour et al., 2006; Wozniak et al., 2006; Wozniak and Levitz, 2008). MoDCs that present *H. capsulatum* antigen can dampen harmful Th2 responses by reducing IL-4 production by CD4^+^ T cells (Szymczak and Deepe, 2010). Additionally, MoDCs can cross-present *H. capsulatum* antigen acquired from apoptotic macrophages to promote CD8^+^ T cell cytotoxic responses under conditions where CD4^+^ T cells are absent or low, as might be found in HIV/AIDS patients (Lin et al., 2005). In vaccination models for *B. dermatitidis* and *H. capsulatum*, robust CD4^+^ T cell priming is dependent on monocyte recruitment to the immunization site (Wuthrich et al., 2012). Human MoDCs exposed to *C. immitis* antigen can induce T cell proliferation and IFNγ secretion (Richards et al., 2001). During respiratory aspergillosis, monocytes differentiate into DCs that traffic *A. fumigatus* conidia to the draining mediastinal lymph nodes and trigger beneficial CD4^+^ T cell responses (Bozza et al., 2002; Hohl et al., 2009). Interestingly, it appears that fungi can also subvert DC antigen presentation pathways. As noted earlier, MoDCs can kill *C. gattii*, but concurrently, the fungus is able to prevent further DC maturation that would lead to a robust adaptive immune response (Huston et al., 2013). Hence, there are multiple steps in antigen presentation by moDCs that can be optimized to generate more effective adaptive immune responses to fungi.

## Trained Immunity: A Second Memory Bank for the Immune System

The adaptive immune response, which includes the generation of memory T and B cells, is the classic mechanism by which the immune system retains memory of foreign antigens to ensure a rapid and specific response upon re-exposure. However, recent studies indicate that monocytes and other innate immune cells can also contribute to immunological memory through the process of trained immunity (Netea et al., 2011, 2016). Trained immunity is established when innate immune cells exposed to microbial antigens undergo sustained epigenetic and metabolic modifications that can enhance their response to a subsequent non-specific stimulus. This innate immune memory is typically maintained over a shorter period of time (weeks to months) compared to adaptive immune memory (years). Notably, the life span of innate immune cells can be quite short, but there is evidence to suggest that hematopoietic stem cells and progenitors of innate immune cells can undergo trained immunity, thereby extending the duration of innate immune memory (Yanez et al., 2013).

Fungal antigens have been found to induce trained immunity in monocytes and their derivative cells. Exposure to β-glucan and to heat-killed or sublethal doses of the commensal fungus *C. albicans* can cause histone modifications and metabolic changes in monocytes and macrophages (Quintin et al., 2012; Saeed et al., 2014). Upon rechallenge with a lethal infection of *C. albicans*, these trained monocytes and macrophages had enhanced cytokine production and improved survival of the infected mice (Browder et al., 1984; Quintin et al., 2012). These host protective effects were confirmed to take place in the absence of T and B cells (Bistoni et al., 1986; Leonhardt et al., 2018). The trained cells also had stronger responses upon restimulation with other microbial antigens and pathogens, including lipopolysaccharide (LPS) and *Staphylococcus aureus* (Di Luzio and Williams, 1978; Quintin et al., 2012; Marakalala et al., 2013). Blocking epigenetic modifications or inhibiting glycolysis can disrupt trained immunity and these beneficial effects (Dominguez-Andres et al., 2017a, 2019). Fungal antigens other than β-glucan may also be able to induce trained immunity. For example, chitin isolated from the commensal yeast *Saccharomyces cerevisiae* can enhance monocyte responses to *C. albicans* as well as gram-positive and gram-negative bacteria (Rizzetto et al., 2016).

There is some evidence that DCs may also have memory-like capabilities. Studies using a vaccine strain of *C. neoformans* indicate that splenic DCs undergo histone modifications that enhance cytokine responses upon rechallenge with a virulent strain of *C. neoformans* (Hole et al., 2019). However, these DCs did not have a robust response to other secondary stimuli including LPS, *S. aureus*, and *C. albicans*. The fungal component of *C. neoformans* that may be involved in stimulating this DC memory also remains to be identified.

Strides are being made to further enhance the effects of trained immunity. For example, deleting SHIP-1 in trained macrophages increases their production of pro-inflammatory cytokines and improves their protection against lethal *C. albicans* infection (Saz-Leal et al., 2018). However, priming innate immune cells with microbial antigens does not always result in beneficial host responses. Trained immunity may play pathologic roles in conditions involving chronic inflammation, and LPS has previously been shown to induce tolerance in monocytes and macrophages to secondary stimuli (Dobrovolskaia and Vogel, 2002; Fan and Cook, 2004; Netea et al., 2016). Thus, as with any immunomodulatory strategies, it will be important to evaluate the full effects of trained immunity on the overall immune response.

## Conclusions and Future Perspectives

The multiple roles of monocytes and their derivative cells in the host response to fungal pathogens highlight their importance in mediating the outcomes of infection. Dissecting the specific mechanisms by which monocytes carry out these functions may enable us to develop novel therapeutics that can target these pathways to improve the mortality rates from invasive fungal infections. With the current intense focus on the role of the microbiome in human health, it will be interesting to further uncover the roles that commensal organisms may play in the trained immunity of monocytes as a key defense mechanism against pathogenic fungi. There is ongoing work to determine whether the heterogeneity of monocyte responses may be tied to their origins in the hematopoietic tissues. For instance, there is evidence that the fate of monocytes is predetermined in the bone marrow and may originate from differences in expression of the transcription factor PU.1, which can dictate their eventual differentiation into iNOS^+^ macrophages vs. moDCs (Menezes et al., 2016). Whether the development of monocytes from different progenitors (i.e., from an MDP vs. GMP progenitor) can influence their ultimate role in the response to fungal pathogens also remains to be determined (Wolf et al., 2019). Besides the pathogens discussed in this review, there are other medically important fungi in which the role of monocytes and monocyte-derived cells is unknown or has only begun to be explored, such as *Pneumocystis jirovecii, Fusarium* spp., the Zygomycetes like *Rhizopus* spp. and *Mucor* spp., and emerging pathogens like *Candida auris* (Friedman and Schwartz, 2019). Therefore, the study of monocytes and immunity to fungal pathogens remains a burgeoning and critical area of research.

## Author Contributions

LH conceptualized, wrote, and edited the manuscript.

### Conflict of Interest

The author declares that the research was conducted in the absence of any commercial or financial relationships that could be construed as a potential conflict of interest.

## References

[B1] Aimanianda V. Bayry J. Bozza S. Kniemeyer O. Perruccio K. Elluru S. R.. (2009). Surface hydrophobin prevents immune recognition of airborne fungal spores. Nature 460, 1117–1121. 10.1038/nature08264 19713928

[B2] Alvarez M. Burn T. Luo Y. Pirofski L. A. Casadevall A. (2009). The outcome of *Cryptococcus neoformans* intracellular pathogenesis in human monocytes. BMC Microbiol. 9:51. 10.1186/1471-2180-9-51 19265539 PMC2670303

[B3] Alvarez M. Casadevall A. (2006). Phagosome extrusion and host-cell survival after *Cryptococcus neoformans* phagocytosis by macrophages. Curr. Biol. 16, 2161–2165. 10.1016/j.cub.2006.09.061 17084702

[B4] Alvarez M. Saylor C. Casadevall A. (2008). Antibody action after phagocytosis promotes *Cryptococcus neoformans* and *Cryptococcus gattii* macrophage exocytosis with biofilm-like microcolony formation. Cell. Microbiol. 10, 1622–1633. 10.1111/j.1462-5822.2008.01152.x 18384661 PMC4294708

[B5] Ampel N. M. Bejarano G. C. Galgiani J. N. (1992). Killing of *Coccidioides immitis* by human peripheral blood mononuclear cells. Infect. Immun. 60, 4200–4204. 10.1128/IAI.60.10.4200-4204.1992 1398931 PMC257453

[B6] Ampel N. M. Galgiani J. N. (1991). Interaction of human peripheral blood mononuclear cells with *Coccidioides immitis* arthroconidia. Cell. Immunol. 133, 253–262. 10.1016/0008-8749(91)90195-h 1991329

[B7] Ampel N. M. Robey I. Nguyen C. T. Roller B. August J. Knox K. S.. (2018). *Ex vivo* cytokine release, determined by a multiplex cytokine assay, in response to coccidioidal antigen stimulation of whole blood among subjects with recently diagnosed primary pulmonary coccidioidomycosis. mSphere 3:e00065-18. 10.1128/mSphere.00065-18 29769377 PMC5956148

[B8] Arango Duque G. Descoteaux A. (2014). Macrophage cytokines: involvement in immunity and infectious diseases. Front. Immunol. 5:491. 10.3389/fimmu.2014.00491 25339958 PMC4188125

[B9] Auffray C. Fogg D. Garfa M. Elain G. Join-Lambert O. Kayal S.. (2007). Monitoring of blood vessels and tissues by a population of monocytes with patrolling behavior. Science 317, 666–670. 10.1126/science.1142883 17673663

[B10] Beaman L. (1991). Effects of recombinant gamma interferon and tumor necrosis factor on *in vitro* interactions of human mononuclear phagocytes with *Coccidioides immitis*. Infect. Immun. 59, 4227–4229. 10.1128/IAI.59.11.4227-4229.1991 1937779 PMC259020

[B11] Beaman L. Benjamini E. Pappagianis D. (1981). Role of lymphocytes in macrophage-induced killing of *Coccidioides immitis in vitro*. Infect. Immun. 34, 347–353. 10.1128/IAI.34.2.347-353.1981 7309228 PMC350872

[B12] Beaman L. Benjamini E. Pappagianis D. (1983). Activation of macrophages by lymphokines: enhancement of phagosome-lysosome fusion and killing of *Coccidioides immitis*. Infect. Immun. 39, 1201–1207. 10.1128/IAI.39.3.1201-1207.1983 6601622 PMC348084

[B13] Beaman L. Holmberg C. A. (1980). *In vitro* response of alveolar macrophages to infection with *Coccidioides immitis*. Infect. Immun. 28, 594–600. 6772563 10.1128/iai.28.2.594-600.1980 PMC550975

[B14] Bistoni F. Vecchiarelli A. Cenci E. Puccetti P. Marconi P. Cassone A. (1986). Evidence for macrophage-mediated protection against lethal *Candida albicans* infection. Infect. Immun. 51, 668–674. 10.1128/IAI.51.2.668-674.1986 3943907 PMC262402

[B15] Bongomin F. Gago S. Oladele R. O. Denning D. W. (2017). Global and multi-national prevalence of fungal diseases-estimate precision. J. Fungi. 3:E57. 10.3390/jof3040057 29371573 PMC5753159

[B16] Bozza S. Gaziano R. Spreca A. Bacci A. Montagnoli C. di Francesco P.. (2002). Dendritic cells transport conidia and hyphae of *Aspergillus fumigatus* from the airways to the draining lymph nodes and initiate disparate Th responses to the fungus. J. Immunol. 168, 1362–1371. 10.4049/jimmunol.168.3.1362 11801677

[B17] Browder I. W. Williams D. L. Kitahama A. Di Luzio N. R. (1984). Modification of post-operative *C. albicans* sepsis by glucan immunostimulation. Int. J. Immunopharmacol. 6, 19–26. 10.1016/0192-0561(84)90030-4 6724765

[B18] Carrion Sde J. Leal S. M. Jr. Ghannoum M. A. Aimanianda V. Latge J. P. Pearlman E. (2013). The RodA hydrophobin on *Aspergillus fumigatus* spores masks dectin-1- and dectin-2-dependent responses and enhances fungal survival *in vivo*. J. Immunol. 191, 2581–2588. 10.4049/jimmunol.1300748 23926321 PMC4020118

[B19] Carson W. E. Ross M. E. Baiocchi R. A. Marien M. J. Boiani N. Grabstein K.. (1995). Endogenous production of interleukin 15 by activated human monocytes is critical for optimal production of interferon-gamma by natural killer cells *in vitro*. J. Clin. Invest. 96, 2578–2582. 10.1172/JCI118321 8675621 PMC185961

[B20] Chang T. H. Huang J. H. Lin H. C. Chen W. Y. Lee Y. H. Hsu L. C.. (2017). Dectin-2 is a primary receptor for NLRP3 inflammasome activation in dendritic cell response to *Histoplasma capsulatum*. PLoS Pathog. 13:e1006485. 10.1371/journal.ppat.1006485 28671985 PMC5510910

[B21] Charlier C. Nielsen K. Daou S. Brigitte M. Chretien F. Dromer F. (2009). Evidence of a role for monocytes in dissemination and brain invasion by *Cryptococcus neoformans*. Infect. Immun. 77, 120–127. 10.1128/IAI.01065-08 18936186 PMC2612285

[B22] Clark C. Drummond R. A. (2019). The hidden cost of modern medical Interventions: how medical advances have shaped the prevalence of human fungal disease. Pathogens 8:E45. 10.3390/pathogens8020045 30987351 PMC6631793

[B23] Cortez K. J. Lyman C. A. Kottilil S. Kim H. S. Roilides E. Yang J.. (2006). Functional genomics of innate host defense molecules in normal human monocytes in response to *Aspergillus fumigatus*. Infect. Immun. 74, 2353–2365. 10.1128/IAI.74.4.2353-2365.2006 16552065 PMC1418921

[B24] Davis M. J. Tsang T. M. Qiu Y. Dayrit J. K. Freij J. B. Huffnagle G. B.. (2013). Macrophage M1/M2 polarization dynamically adapts to changes in cytokine microenvironments in *Cryptococcus neoformans* infection. MBio 4, e00264–e00213. 10.1128/mBio.00264-13 23781069 PMC3684832

[B25] del Pilar Jimenez A. M. Viriyakosol S. Walls L. Datta S. K. Kirkland T. Heinsbroek S. E. (2008). Susceptibility to *Coccidioides* species in C57BL/6 mice is associated with expression of a truncated splice variant of Dectin-1 (Clec7a). Genes. Immun. 9, 338–348. 10.1038/gene.2008.23 18418396 PMC3681288

[B26] Di Luzio N. R. Williams D. L. (1978). Protective effect of glucan against systemic *Staphylococcus aureus* septicemia in normal and leukemic mice. Infect. Immun. 20, 804–810. 10.1128/IAI.20.3.804-810.1978 352959 PMC421929

[B27] Dobrovolskaia M. A. Vogel S. N. (2002). Toll receptors, CD14, and macrophage activation and deactivation by LPS. Microbes. Infect. 4, 903–914. 10.1016/s1286-4579(02)01613-1 12106783

[B28] Dominguez-Andres J. Arts R. J. W. Ter Horst R. Gresnigt M. S. Smeekens S. P. Ratter J. M.. (2017a). Rewiring monocyte glucose metabolism via C-type lectin signaling protects against disseminated candidiasis. PLoS Pathog. 13:e1006632. 10.1371/journal.ppat.1006632 28922415 PMC5619837

[B29] Dominguez-Andres J. Feo-Lucas L. Minguito de la Escalera M. Gonzalez L. Lopez-Bravo M. Ardavin C. (2017b). Inflammatory Ly6C (high) monocytes protect against candidiasis through IL-15-driven NK cell/neutrophil activation. Immunity 46, 1059–1072 e1054. 10.1016/j.immuni.2017.05.009 28636955

[B30] Dominguez-Andres J. Ferreira A. V. Jansen T. Smithers N. Prinjha R. K. Furze R. C.. (2019). Bromodomain inhibitor I-BET151 suppresses immune responses during fungal-immune interaction. Eur. J. Immunol. 49, 2044–2050 10.1002/eji.201848081 31206650 PMC6899658

[B31] d'Ostiani C. F. Del Sero G. Bacci A. Montagnoli C. Spreca A. Mencacci A.. (2000). Dendritic cells discriminate between yeasts and hyphae of the fungus *Candida albicans*. Implications for initiation of T helper cell immunity *in vitro* and *in vivo*. J. Exp. Med. 191, 1661–1674. 10.1084/jem.191.10.1661 10811860 PMC2193147

[B32] Eisenbarth S. C. (2019). Dendritic cell subsets in T cell programming: location dictates function. Nat. Rev. Immunol. 19, 89–103. 10.1038/s41577-018-0088-1 30464294 PMC7755085

[B33] Eissenberg L. G. Goldman W. E. Schlesinger P. H. (1993). *Histoplasma capsulatum* modulates the acidification of phagolysosomes. J. Exp. Med. 177, 1605–1611. 10.1084/jem.177.6.1605 8496679 PMC2191039

[B34] Erwig L. P. Gow N. A. (2016). Interactions of fungal pathogens with phagocytes. Nat. Rev. Microbiol. 14, 163–176. 10.1038/nrmicro.2015.21 26853116

[B35] Espinosa V. Jhingran A. Dutta O. Kasahara S. Donnelly R. Du P.. (2014). Inflammatory monocytes orchestrate innate antifungal immunity in the lung. PLoS Pathog. 10:e1003940. 10.1371/journal.ppat.1003940 24586155 PMC3930594

[B36] Fan H. Cook J. A. (2004). Molecular mechanisms of endotoxin tolerance. J. Endotoxin. Res. 10, 71–84. 10.1179/096805104225003997 15119998

[B37] Feldmesser M. Kress Y. Novikoff P. Casadevall A. (2000). *Cryptococcus neoformans* is a facultative intracellular pathogen in murine pulmonary infection. Infect. Immun. 68, 4225–4237. 10.1128/iai.68.7.4225-4237.2000 10858240 PMC101732

[B38] Fierer J. (2007). The role of IL-10 in genetic susceptibility to coccidioidomycosis on mice. Ann. N.Y. Acad. Sci. 1111, 236–244. 10.1196/annals.1406.048 17363443

[B39] Friedman D. Z. P. Schwartz I. S. (2019). Emerging fungal infections: new patients, new patterns, and new pathogens. J. Fungi 5:E67. 10.3390/jof5030067 31330862 PMC6787706

[B40] Gantner B. N. Simmons R. M. Underhill D. M. (2005). Dectin-1 mediates macrophage recognition of *Candida albicans* yeast but not filaments. EMBO J. 24, 1277–1286. 10.1038/sj.emboj.7600594 15729357 PMC556398

[B41] Gersuk G. M. Underhill D. M. Zhu L. Marr K. A. (2006). Dectin-1 and TLRs permit macrophages to distinguish between different *Aspergillus fumigatus* cellular states. J. Immunol. 176, 3717–3724. 10.4049/jimmunol.176.6.3717 16517740

[B42] Gildea L. A. Morris R. E. Newman S. L. (2001). *Histoplasma capsulatum* yeasts are phagocytosed via very late antigen-5, killed, and processed for antigen presentation by human dendritic cells. J. Immunol. 166, 1049–1056. 10.4049/jimmunol.166.2.1049 11145684

[B43] Ginhoux F. Guilliams M. (2016). Tissue-resident macrophage ontogeny and homeostasis. Immunity 44, 439–449. 10.1016/j.immuni.2016.02.024 26982352

[B44] Gow N. A. van de Veerdonk F. L. Brown A. J. Netea M. G. (2011). *Candida albicans* morphogenesis and host defence: discriminating invasion from colonization. Nat. Rev. Microbiol. 10, 112–122. 10.1038/nrmicro2711 22158429 PMC3624162

[B45] Grimm M. J. Vethanayagam R. R. Almyroudis N. G. Dennis C. G. Khan A. N. D'Auria A. C.. (2013). Monocyte- and macrophage-targeted NADPH oxidase mediates antifungal host defense and regulation of acute inflammation in mice. J. Immunol. 190, 4175–4184. 10.4049/jimmunol.1202800 23509361 PMC3622122

[B46] Hachem R. Gomes M. Z. El Helou G. El Zakhem A. Kassis C. Ramos E.. (2014). Invasive aspergillosis caused by *Aspergillus terreus*: an emerging opportunistic infection with poor outcome independent of azole therapy. J. Antimicrob. Chemother. 69, 3148–3155. 10.1093/jac/dku241 25006241

[B47] Hardison S. E. Herrera G. Young M. L. Hole C. R. Wozniak K. L. Wormley F. L. Jr. (2012). Protective immunity against pulmonary cryptococcosis is associated with STAT1-mediated classical macrophage activation. J. Immunol. 189, 4060–4068. 10.4049/jimmunol.1103455 22984078 PMC3466339

[B48] Heitman J. Kozel T. R. Kwon-Chung K. J. Perfect J. R. Casadevall A. (2010). Cryptococcus: From Human Pathogen to Model Yeast. Washington, DC: ASM Press 10.1128/9781555816858

[B49] Heung L. J. Hohl T. M. (2019). Inflammatory monocytes are detrimental to the host immune response during acute infection with *Cryptococcus neoformans*. PLoS Pathog. 15:e1007627. 10.1371/journal.ppat.1007627 30897162 PMC6428256

[B50] Hoeffel G. Ginhoux F. (2018). Fetal monocytes and the origins of tissue-resident macrophages. Cell. Immunol. 330, 5–15. 10.1016/j.cellimm.2018.01.001 29475558

[B51] Hohl T. M. Rivera A. Lipuma L. Gallegos A. Shi C. Mack M.. (2009). Inflammatory monocytes facilitate adaptive CD4 T cell responses during respiratory fungal infection. Cell Host Microbe 6, 470–481. 10.1016/j.chom.2009.10.007 19917501 PMC2785497

[B52] Hohl T. M. Van Epps H. L. Rivera A. Morgan L. A. Chen P. L. Feldmesser M.. (2005). *Aspergillus fumigatus* triggers inflammatory responses by stage-specific beta-glucan display. PLoS Pathog. 1:e30. 10.1371/journal.ppat.0010030 16304610 PMC1287910

[B53] Hole C. R. Wager C. M. L. Castro-Lopez N. Campuzano A. Cai H. Wozniak K. L.. (2019). Induction of memory-like dendritic cell responses *in vivo*. Nat. Commun. 10:2955. 10.1038/s41467-019-10486-5 31273203 PMC6609631

[B54] Howard D. H. (1964). Intracellular behavior of *Histoplasma capsulatum*. J. Bacteriol. 87, 33–38. 10.1128/JB.87.1.33-38.1964 14102870 PMC276957

[B55] Hsieh S. H. Kurzai O. Brock M. (2017). Persistence within dendritic cells marks an antifungal evasion and dissemination strategy of *Aspergillus terreus*. Sci. Rep. 7:10590. 10.1038/s41598-017-10914-w 28878289 PMC5587622

[B56] Huang J. H. Lin C. Y. Wu S. Y. Chen W. Y. Chu C. L. Brown G. D.. (2015). CR3 and Dectin-1 collaborate in macrophage cytokine response through association on lipid rafts and activation of Syk-JNK-AP-1 pathway. PLoS Pathog. 11:e1004985. 10.1371/journal.ppat.1004985 26132276 PMC4488469

[B57] Huston S. M. Li S. S. Stack D. Timm-McCann M. Jones G. J. Islam A.. (2013). *Cryptococcus gattii* is killed by dendritic cells, but evades adaptive immunity by failing to induce dendritic cell maturation. J. Immunol. 191, 249–261. 10.4049/jimmunol.1202707 23740956

[B58] Jaeger M. Matzaraki V. Aguirre-Gamboa R. Gresnigt M. S. Chu X. Johnson M. D.. (2019). A genome-wide functional genomics approach identifies susceptibility pathways to fungal bloodstream infection in humans. J. Infect. Dis. 220, 862–872. 10.1093/infdis/jiz206 31241743 PMC6667794

[B59] Jakubzick C. V. Randolph G. J. Henson P. M. (2017). Monocyte differentiation and antigen-presenting functions. Nat. Rev. Immunol. 17, 349–362. 10.1038/nri.2017.28 28436425

[B60] Jimenez Mdel P. Walls L. Fierer J. (2006). High levels of interleukin-10 impair resistance to pulmonary coccidioidomycosis in mice in part through control of nitric oxide synthase 2 expression. Infect. Immun. 74, 3387–3395. 10.1128/IAI.01985-05 16714569 PMC1479230

[B61] Joly S. Ma N. Sadler J. J. Soll D. R. Cassel S. L. Sutterwala F. S. (2009). Cutting edge: *Candida albicans* hyphae formation triggers activation of the Nlrp3 inflammasome. J. Immunol. 183, 3578–3581. 10.4049/jimmunol.0901323 19684085 PMC2739101

[B62] Kim H. S. Choi E. H. Khan J. Roilides E. Francesconi A. Kasai M.. (2005). Expression of genes encoding innate host defense molecules in normal human monocytes in response to *Candida albicans*. Infect. Immun. 73, 3714–3724. 10.1128/IAI.73.6.3714-3724.2005 15908401 PMC1111842

[B63] Lawrence T. Natoli G. (2011). Transcriptional regulation of macrophage polarization: enabling diversity with identity. Nat. Rev. Immunol. 11, 750–761. 10.1038/nri3088 22025054

[B64] Leonhardt J. Grosse S. Marx C. Siwczak F. Stengel S. Bruns T.. (2018). *Candida albicans* beta-glucan differentiates human monocytes into a specific subset of macrophages. Front. Immunol. 9:2818. 10.3389/fimmu.2018.02818 30555483 PMC6284042

[B65] Lin J. S. Huang J. H. Hung L. Y. Wu S. Y. Wu-Hsieh B. A. (2010). Distinct roles of complement receptor 3, Dectin-1, and sialic acids in murine macrophage interaction with *Histoplasma* yeast. J. Leukoc. Biol. 88, 95–106. 10.1189/jlb.1109717 20360401

[B66] Lin J. S. Yang C. W. Wang D. W. Wu-Hsieh B. A. (2005). Dendritic cells cross-present exogenous fungal antigens to stimulate a protective CD8 T cell response in infection by *Histoplasma capsulatum*. J. Immunol. 174, 6282–6291. 10.4049/jimmunol.174.10.6282 15879127

[B67] Lionakis M. S. Iliev I. D. Hohl T. M. (2017). Immunity against fungi. JCI Insight 2:e93156. 10.1172/jci.insight.93156 28570272 PMC5453709

[B68] Ma H. Croudace J. E. Lammas D. A. May R. C. (2006). Expulsion of live pathogenic yeast by macrophages. Curr. Biol. 16, 2156–2160. 10.1016/j.cub.2006.09.032 17084701

[B69] Mansour M. K. Latz E. Levitz S. M. (2006). *Cryptococcus neoformans* glycoantigens are captured by multiple lectin receptors and presented by dendritic cells. J. Immunol. 176, 3053–3061. 10.4049/jimmunol.176.5.3053 16493064

[B70] Marakalala M. J. Williams D. L. Hoving J. C. Engstad R. Netea M. G. Brown G. D. (2013). Dectin-1 plays a redundant role in the immunomodulatory activities of beta-glucan-rich ligands *in vivo*. Microbes. Infect. 15, 511–515. 10.1016/j.micinf.2013.03.002 23518266 PMC3839404

[B71] Martinez F. O. Gordon S. (2014). The M1 and M2 paradigm of macrophage activation: time for reassessment. F1000Prime Rep. 6:13. 10.12703/P6-13 24669294 PMC3944738

[B72] Menezes S. Melandri D. Anselmi G. Perchet T. Loschko J. Dubrot J. (2016). The heterogeneity of Ly6C(hi) monocytes controls their differentiation into iNOS(+) macrophages or monocyte-derived dendritic cells. Immunity 45, 1205–1218. 10.1016/j.immuni.2016.12.001 28002729 PMC5196026

[B73] Mosser D. M. Edwards J. P. (2008). Exploring the full spectrum of macrophage activation. Nat. Rev. Immunol. 8, 958–969. 10.1038/nri2448 19029990 PMC2724991

[B74] Muller U. Stenzel W. Piehler D. Grahnert A. Protschka M. Kohler G.. (2013). Abrogation of IL-4 receptor-α-dependent alternatively activated macrophages is sufficient to confer resistance against pulmonary cryptococcosis despite an ongoing T(h)2 response. Int. Immunol. 25, 459–470. 10.1093/intimm/dxt003 23532373

[B75] Murray P. J. (2018). Immune regulation by monocytes. Semin. Immunol. 35, 12–18. 10.1016/j.smim.2017.12.005 29290545

[B76] Murray P. J. Allen J. E. Biswas S. K. Fisher E. A. Gilroy D. W. Goerdt S.. (2014). Macrophage activation and polarization: nomenclature and experimental guidelines. Immunity 41, 14–20. 10.1016/j.immuni.2014.06.008 25035950 PMC4123412

[B77] Netea M. G. Joosten L. A. Latz E. Mills K. H. Natoli G. Stunnenberg H. G.. (2016). Trained immunity: a program of innate immune memory in health and disease. Science 352:aaf1098. 10.1126/science.aaf1098 27102489 PMC5087274

[B78] Netea M. G. Quintin J. van der Meer J. W. (2011). Trained immunity: a memory for innate host defense. Cell. Host. Microbe. 9, 355–361. 10.1016/j.chom.2011.04.006 21575907

[B79] Newman S. L. Gootee L. Bucher C. Bullock W. E. (1991). Inhibition of intracellular growth of *Histoplasma capsulatum* yeast cells by cytokine-activated human monocytes and macrophages. Infect. Immun. 59, 737–741. 10.1128/IAI.59.2.737-741.1991 1898916 PMC257824

[B80] Newman S. L. Gootee L. Kidd C. Ciraolo G. M. Morris R. (1997). Activation of human macrophage fungistatic activity against *Histoplasma capsulatum* upon adherence to type 1 collagen matrices. J. Immunol. 158, 1779–1786. 9029116

[B81] Ngo L. Y. Kasahara S. Kumasaka D. K. Knoblaugh S. E. Jhingran A. Hohl T. M. (2014). Inflammatory monocytes mediate early and organ-specific innate defense during systemic candidiasis. J. Infect. Dis. 209, 109–119. 10.1093/infdis/jit413 23922372 PMC3864383

[B82] Nicola A. M. Robertson E. J. Albuquerque P. Derengowski Lda S. Casadevall A. (2011). Nonlytic exocytosis of *Cryptococcus neoformans* from macrophages occurs *in vivo* and is influenced by phagosomal pH. MBio 2:e00167-11. 10.1128/mBio.00167-11 21828219 PMC3150755

[B83] Noble S. M. Gianetti B. A. Witchley J. N. (2017). *Candida albicans* cell-type switching and functional plasticity in the mammalian host. Nat. Rev. Microbiol. 15, 96–108. 10.1038/nrmicro.2016.157 27867199 PMC5957277

[B84] Olingy C. E. Dinh H. Q. Hedrick C. C. (2019). Monocyte heterogeneity and functions in cancer. J. Leukoc. Biol. 106, 309–322. 10.1002/JLB.4RI0818-311R 30776148 PMC6658332

[B85] Osterholzer J. J. Chen G. H. Olszewski M. A. Curtis J. L. Huffnagle G. B. Toews G. B. (2009). Accumulation of CD11b+ lung dendritic cells in response to fungal infection results from the CCR2-mediated recruitment and differentiation of Ly-6Chigh monocytes. J. Immunol. 183, 8044–8053. 10.4049/jimmunol.0902823 19933856 PMC4043300

[B86] Osterholzer J. J. Chen G. H. Olszewski M. A. Zhang Y. M. Curtis J. L. Huffnagle G. B.. (2011). Chemokine receptor 2-mediated accumulation of fungicidal exudate macrophages in mice that clear cryptococcal lung infection. Am. J. Pathol. 178, 198–211. 10.1016/j.ajpath.2010.11.006 21224057 PMC3069860

[B87] Osterholzer J. J. Curtis J. L. Polak T. Ames T. Chen G. H. McDonald R.. (2008). CCR2 mediates conventional dendritic cell recruitment and the formation of bronchovascular mononuclear cell infiltrates in the lungs of mice infected with *Cryptococcus neoformans*. J. Immunol. 181, 610–620. 10.4049/jimmunol.181.1.610 18566428 PMC2735104

[B88] Pfaller M. A. Diekema D. J. (2010). Epidemiology of invasive mycoses in North America. Crit. Rev. Microbiol. 36, 1–53. 10.3109/10408410903241444 20088682

[B89] Quintin J. Saeed S. Martens J. H. A. Giamarellos-Bourboulis E. J. Ifrim D. C. Logie C.. (2012). *Candida albicans* infection affords protection against reinfection via functional reprogramming of monocytes. Cell Host Microbe. 12, 223–232. 10.1016/j.chom.2012.06.006 22901542 PMC3864037

[B90] Richards J. O. Ampel N. M. Galgiani J. N. Lake D. F. (2001). Dendritic cells pulsed with *Coccidioides immitis* lysate induce antigen-specific naive T cell activation. J. Infect. Dis. 184, 1220–1224. 10.1086/323664 11598850

[B91] Rivera A. Hohl T. M. Collins N. Leiner I. Gallegos A. Saijo S.. (2011). Dectin-1 diversifies *Aspergillus fumigatus*-specific T cell responses by inhibiting T helper type 1 CD4 T cell differentiation. J. Exp. Med. 208, 369–381. 10.1084/jem.20100906 21242294 PMC3039849

[B92] Rivera A. Van Epps H. L. Hohl T. M. Rizzuto G. Pamer E. G. (2005). Distinct CD4+-T-cell responses to live and heat-inactivated *Aspergillus fumigatus* conidia. Infect. Immun. 73, 7170–7179. 10.1128/IAI.73.11.7170-7179.2005 16239511 PMC1273880

[B93] Rizzetto L. Ifrim D. C. Moretti S. Tocci N. Cheng S. C. Quintin J.. (2016). Fungal chitin induces trained immunity in human monocytes during cross-talk of the host with *Saccharomyces cerevisiae*. J. Biol. Chem. 291, 7961–7972. 10.1074/jbc.M115.699645 26887946 PMC4825003

[B94] Rocco N. M. Carmen J. C. Klein B. S. (2011). *Blastomyces dermatitidis* yeast cells inhibit nitric oxide production by alveolar macrophage inducible nitric oxide synthase. Infect. Immun. 79, 2385–2395. 10.1128/IAI.01249-10 21444664 PMC3125838

[B95] Roy R. M. Klein B. S. (2012). Dendritic cells in antifungal immunity and vaccine design. Cell. Host. Microbe. 11, 436–446. 10.1016/j.chom.2012.04.005 22607797 PMC3401965

[B96] Saeed S. Quintin J. Kerstens H. H. Rao N. A. Aghajanirefah A. Matarese F.. (2014). Epigenetic programming of monocyte-to-macrophage differentiation and trained innate immunity. Science 345:1251086. 10.1126/science.1251086 25258085 PMC4242194

[B97] Saz-Leal P. Del Fresno C. Brandi P. Martinez-Cano S. Dungan O. M. Chisholm J. D.. (2018). Targeting SHIP-1 in myeloid cells enhances trained immunity and boosts response to infection. Cell. Rep. 25, 1118–1126. 10.1016/j.celrep.2018.09.092 30380404 PMC6226423

[B98] Shi C. Pamer E. G. (2011). Monocyte recruitment during infection and inflammation. Nat. Rev. Immunol. 11, 762–774. 10.1038/nri3070 21984070 PMC3947780

[B99] Slesiona S. Gressler M. Mihlan M. Zaehle C. Schaller M. Barz D.. (2012). Persistence versus escape: *Aspergillus terreus* and *Aspergillus fumigatus* employ different strategies during interactions with macrophages. PLoS ONE 7:e31223. 10.1371/journal.pone.0031223 22319619 PMC3272006

[B100] Steele C. Rapaka R. R. Metz A. Pop S. M. Williams D. L. Gordon S.. (2005). The beta-glucan receptor dectin-1 recognizes specific morphologies of *Aspergillus fumigatus*. PLoS Pathog, 1:e42. 10.1371/journal.ppat.0010042 16344862 PMC1311140

[B101] Stenzel W. Muller U. Kohler G. Heppner F. L. Blessing M. McKenzie A. N. (2009). IL-4/IL-13-dependent alternative activation of macrophages but not microglial cells is associated with uncontrolled cerebral cryptococcosis. Am. J. Pathol. 174, 486–496. 10.2353/ajpath.2009.080598 19147811 PMC2630557

[B102] Sterkel A. K. Mettelman R. Wuthrich M. Klein B. S. (2015). The unappreciated intracellular lifestyle of *Blastomyces dermatitidis*. J. Immunol. 194, 1796–1805. 10.4049/jimmunol.1303089 25589071 PMC4373353

[B103] Subramanian Vignesh K. Landero Figueroa J. A. Porollo A. Caruso J. A. Deepe G. S. Jr. (2013). Granulocyte macrophage-colony stimulating factor induced Zn sequestration enhances macrophage superoxide and limits intracellular pathogen survival. Immunity 39, 697–710. 10.1016/j.immuni.2013.09.006 24138881 PMC3841917

[B104] Syme R. M. Spurrell J. C. Amankwah E. K. Green F. H. Mody C. H. (2002). Primary dendritic cells phagocytose *Cryptococcus neoformans* via mannose receptors and Fcgamma receptor II for presentation to T lymphocytes. Infect. Immun. 70, 5972–5981. 10.1128/iai.70.11.5972-5981.2002 12379672 PMC130340

[B105] Szymczak W. A. Deepe G. S. Jr. (2009). The CCL7-CCL2-CCR2 axis regulates IL-4 production in lungs and fungal immunity. J. Immunol. 183, 1964–1974. 10.4049/jimmunol.0901316 19587014 PMC2743878

[B106] Szymczak W. A. Deepe G. S. Jr. (2010). Antigen-presenting dendritic cells rescue CD4-depleted CCR2-/- mice from lethal *Histoplasma capsulatum* infection. Infect. Immun. 78, 2125–2137. 10.1128/IAI.00065-10 20194586 PMC2863525

[B107] Thompson A. Davies L. C. Liao C. T. da Fonseca D. M. Griffiths J. S. Andrews R.. (2019). The protective effect of inflammatory monocytes during systemic *C. albicans* infection is dependent on collaboration between C-type lectin-like receptors. PLoS Pathog. 15:e1007850. 10.1371/journal.ppat.1007850 31242262 PMC6594653

[B108] Traynor T. R. Kuziel W. A. Toews G. B. Huffnagle G. B. (2000). CCR2 expression determines T1 versus T2 polarization during pulmonary *Cryptococcus neoformans* infection. J. Immunol. 164, 2021–2027. 10.4049/jimmunol.164.4.2021 10657654

[B109] Uribe-Querol E. Rosales C. (2017). Control of phagocytosis by microbial pathogens. Front. Immunol. 8:1368. 10.3389/fimmu.2017.01368 29114249 PMC5660709

[B110] Viriyakosol S. Fierer J. Brown G. D. Kirkland T. N. (2005). Innate immunity to the pathogenic fungus *Coccidioides posadasii* is dependent on Toll-like receptor 2 and Dectin-1. Infect. Immun. 73, 1553–1560. 10.1128/IAI.73.3.1553-1560.2005 15731053 PMC1064940

[B111] Viriyakosol S. Jimenez Mdel P. Gurney M. A. Ashbaugh M. E. Fierer J. (2013). Dectin-1 is required for resistance to coccidioidomycosis in mice. MBio 4, e00597–e00512. 10.1128/mBio.00597-12 23386437 PMC3562125

[B112] Viriyakosol S. Walls L. Okamoto S. Raz E. Williams D. L. Fierer J. (2018). Myeloid Differentiation Factor 88 and Interleukin-1R1 signaling contribute to resistance to *Coccidioides immitis*. Infect. Immun. 86:e00028-18. 10.1128/IAI.00028-18 29610256 PMC5964525

[B113] Whitney P. G. Bar E. Osorio F. Rogers N. C. Schraml B. U. Deddouche S.. (2014). Syk signaling in dendritic cells orchestrates innate resistance to systemic fungal infection. PLoS Pathog. 10:e1004276. 10.1371/journal.ppat.1004276 25033445 PMC4102599

[B114] Wolf A. A. Yanez A. Barman P. K. Goodridge H. S. (2019). The ontogeny of monocyte subsets. Front. Immunol. 10:1642. 10.3389/fimmu.2019.01642 31379841 PMC6650567

[B115] Wozniak K. L. Levitz S. M. (2008). *Cryptococcus neoformans* enters the endolysosomal pathway of dendritic cells and is killed by lysosomal components. Infect. Immun. 76, 4764–4771. 10.1128/IAI.00660-08 18678670 PMC2546838

[B116] Wozniak K. L. Vyas J. M. Levitz S. M. (2006). *In vivo* role of dendritic cells in a murine model of pulmonary cryptococcosis. Infect. Immun. 74, 3817–3824. 10.1128/IAI.00317-06 16790753 PMC1489690

[B117] Wu-Hsieh B. A. Howard D. H. (1987). Inhibition of the intracellular growth of *Histoplasma capsulatum* by recombinant murine gamma interferon. Infect. Immun. 55, 1014–1016. 10.1128/IAI.55.4.1014-1016.1987 3104206 PMC260455

[B118] Wuthrich M. Ersland K. Sullivan T. Galles K. Klein B. S. (2012). Fungi subvert vaccine T cell priming at the respiratory mucosa by preventing chemokine-induced influx of inflammatory monocytes. Immunity 36, 680–692. 10.1016/j.immuni.2012.02.015 22483803 PMC3334432

[B119] Yanez A. Coetzee S. G. Olsson A. Muench D. E. Berman B. P. Hazelett D. J.. (2017). Granulocyte-monocyte progenitors and monocyte-dendritic cell progenitors independently produce functionally distinct monocytes. Immunity 47, 890–902.e894. 10.1016/j.immuni.2017.10.021 29166589 PMC5726802

[B120] Yanez A. Hassanzadeh-Kiabi N. Ng M. Y. Megias J. Subramanian A. Liu G. Y.. (2013). Detection of a TLR2 agonist by hematopoietic stem and progenitor cells impacts the function of the macrophages they produce. Eur. J. Immunol. 43, 2114–2125. 10.1002/eji.201343403 23661549 PMC3783206

[B121] Yona S. Kim K. W. Wolf Y. Mildner A. Varol D. Breker M.. (2013). Fate mapping reveals origins and dynamics of monocytes and tissue macrophages under homeostasis. Immunity 38, 79–91. 10.1016/j.immuni.2012.12.001 23273845 PMC3908543

[B122] Zhang X. He D. Gao S. Wei Y. Wang L. (2019). *Aspergillus fumigatus* enhances human NK cell activity by regulating M1 macrophage polarization. Mol. Med. Rep. 20, 1241–1249. 10.3892/mmr.2019.10365 31173233 PMC6625407

